# Psychometric Properties and Factor Structure of the French Version of the Behavioral Activation for Depression Scale (BADS) in Non-Clinical Adults

**DOI:** 10.5334/pb.542

**Published:** 2021-02-01

**Authors:** Audrey Krings, Catherine Bortolon, Hanan Yazbek, Sylvie Blairy

**Affiliations:** 1 Psychology and Neurosciences of Cognition, University of Liège, Place des Orateurs, 1, 4000 Liège, Belgium; 2 Laboratoire Inter-universitaire de Psychologie: Personnalité, Cognition et Changement Social, Université Grenoble Alpes, 1251 avenue Centrale, 8058 Grenoble Cedex 9, France; 3 Département de Psychiatrie adulte, Hôpital La Colombière – Centre Hospitalier Universitaire de Montpellier, Montpellier, France

**Keywords:** Behavioral activation, psychometric properties, depression

## Abstract

Behavioral activation (BA) is a well-established empirical treatment for depression that aims to improve depressive mood by increasing activation and reducing avoidance. Therefore, it is essential to evaluate activation and avoidance when a BA treatment is applied. The Behavioral Activation for Depression Scale (BADS) was developed to measure the changes in activation and avoidance over the course of BA treatment of depression. This study aims to validate the French version of this scale. In a first study, 131 bilingual adults were recruited to explored internal consistency, test-retest reliability and construct validity of the final French version. In a second study, 409 non-clinical adults completed an online survey assessing concurrent measures. Results of the first study suggested good internal consistency, test-retest reliability and construct validity. The second study revealed a confirmatory factor analysis supporting the original four-factor structure, with Activation, Avoidance/Rumination, Work/School Impairment, and Social Impairment subscales. Results also revealed that a 5-factor model distinguishing Behavioral Avoidance and Rumination had a better fit than the original four-factor structure. All subscales showed adequate internal consistency and good construct validity with evidence of convergent validity with depressive symptoms, brooding, psychological flexibility, negative automatic thought, behavioral inhibition and activation system. Furthermore, the French BADS total scale and subscales showed a good ability to predict depressive symptoms. The French version of the BADS appears to be a reliable tool for clinician and researchers to assess mechanisms of change in BA interventions.

## INTRODUCTION

Major depressive disorder is one of the most common mental disorders, and one of the leading causes of disability worldwide (Kessler & Bromet, 2013). It has a lifetime prevalence of 10,8 % (Lim et al., 2018). The World Health Organization estimates that depression and anxiety are common mental disorders in Europe with around 25% of the population affected if moderate forms of depression are considered. Behavioral theories postulate that depressive symptoms are produced and maintained by a decrease in the level of response-contingent positive reinforcement (RCPR), defined as positive or pleasurable consequences of individual’s behaviors that increase the likelihood that those behaviors will happen again (Lewinsohn, 1974; Lewinsohn & Graf, 1973). Studies have revealed that a low level of RCPR in depressed patients is related to decreased involvement in pleasurable and reinforcing activities and a withdrawal from professional or social activities (Carvalho & Hopko, 2011; Hopko, Lejuez, Ruggiero, & Eifert, 2003). A lower rate of RCPR can be explained by both decreased activation, that is, a reduction in the degree of focused, goal-directed engagement in activities, and increased avoidance, which is defined as the tendency to avoid negative aversive states that can, ultimately, result in limited exposure to potentially rewarding activities. Thus, both of these phenomena are involved in the development and maintenance of depressive symptoms (Chen, Liu, Rapee, & Pillay, 2013; Collado, Castillo, Maero, Lejuez, & MacPherson, 2014; Wagener, Baeyens, & Blairy, 2016).

Based on this behavioral theory of depression, many researchers have proposed that an intervention based on behavioral activation could be a valuable alternative treatment for depression. Indeed, Behavioral activation (BA) treatment is now a well-established empirical treatment for depression with medium to large effect size (see meta-analyses Cuipers, et al., 2007; Ekers et al., 2014; Mazzuchelli et al., 2009). BA aims to increase activation and reduce avoidance in order to enhance the level of RCPR and consequently reduce depressive affects (Cuijpers, van Straten, & Warmerdam, 2007; Lejuez, Hopko, LePage, Hopko, & McNeil, 2001; Manos, Kanter, & Busch, 2010). Measuring activation and avoidance when using BA treatment is important to understand the mechanisms of change leading to an improvement in depressive symptoms. The Behavioral Activation for Depression Scale (BADS) was developed by Kanter’s team to measure activation, avoidance and the related impairments (Kanter, Mulick, Busch, Berlin, & Martell, 2007). This scale has since been translated into German and Dutch (Fuhr, Hautzinger, Krisch, Berking, & Ebert, 2016; Kanter, Rusch, Busch, & Sedivy, 2008; Kanter et al., 2007; Raes, Hoes, Van Gucht, Kanter, & Hermans, 2010; Teismann, Ertle, Furka, Willutzki, & Hoyer, 2016).

The BADS is 25-item self-report scale comprising four subscales. The first subscale, ‘Activation’, measures focused, goal-directed action and completion of planned activities (e.g., ‘I did something that was hard to do but it was worth it’). The second subscale, ‘Avoidance/Rumination’, measures the avoidance of negative aversive states and engagement in brooding rumination (e.g., ‘I tried not to think about certain things’ or ‘I stayed in bed for too long even though I had things to do’). The third factor, ‘Work/School Impairment’, measures the consequences of inactivity for work and school responsibilities (e.g., ‘I took time off of work/school/work/chores/responsibilities simply because I was not as active as I needed to be’). The fourth subscale, ‘Social Impairment’, measures the consequences of inactivity for social contact (e.g., ‘I pushed people away with my negativity’).

A short form of the BADS already exists in French to measure activation and avoidance. However, this scale does not take into account impairments in different areas of life (e.g., work, school or social impairments), and these measures can be interesting. For example, using the long form of the BADS, Renner, Ji, Pictet, Holmes, and Blackwell (2017) investigated the effects of positive mental imagery of future events on behavioral activation in depression (Renner et al., 2017). Their results revealed an improvement in the experimental group’s Social Impairments score. The use of the short form of this scale would not have allowed the team to identify this change. Furthermore, apart from the severity of activation and avoidance, measuring the consequences for life functioning is relevant because the negative impact on an individual’s functioning is a critical aspect of the diagnosis of depression and thus is relevant for clinical practice. Finally, it is essential to note that the code of ethics of Belgian psychologists recommends evaluating the efficiency of treatment provided in order to recognize any potentially harmful and foreseeable consequences of their work in time (Chap. IV, Sec. III, Art. 31).

In this study, the long-form BADS was translated into French to provide a validated measure of changes in activation and avoidance and the associated impairments for clinicians and researchers. According to this aim, a first study explored internal consistency, test-retest reliability and construct validity of the final French version with a bilingual sample. A second study submitted the final scale to a confirmatory factor analysis (CFA) in a non-clinical adult sample to replicate the original factor structure. Then, this second study submitted the scale to the analysis of construct validity through comparisons with other concurrent measures that assess partially-overlapping constructs as well as with multiple linear regressions.

## PRELIMINARY SCALE TRANSLATION

The original instrument was first translated into French by two independent bilingual adults.^1^ According to the transcultural translation procedure (Vallerand, 1989), items were first translated into French by the first author and back-translated into English by a bilingual expert. A committee approach was used to compare the two versions. Discrepancies between the two versions were discussed, and adjustments were made to the French version. The committee process was intercultural and involved Belgian-French collaboration.

To test the pre-final version of the instrument, a cognitive debriefing was conducted with a monolingual sample (Sousa & Rojjanasrirat, 2011; Touzani & Salaani, 2000). Fifty psychology students were asked to rate the clarity of the instructions, the items and the response format of this version using a dichotomous scale (clear or unclear). If anything was rated as unclear, participants were asked to provide suggestions as how to rewrite it to make it clearer. Instructions, response format and items that were found to be unclear by at least 20% of the sample were rewritten with a committee approach as well. To further determine the conceptual and content equivalence of the items, 10 experts (psychology researchers at Liege University) who were informed about the content areas of the instrument undertook to follow similar instructions. In addition, they were asked to evaluate the relevance of the scale’s content-related validity (from not relevant to very relevant) (Touzani & Salaani, 2000). Adjustments were then made and a final French version was developed (see Appendix 1).

## STUDY 1

The first study was conducted to measure the preliminary psychometric properties of this final version. We used an approach proposed by Haccoun (Haccoun, 1987) to test internal consistency, temporal stability, and construct validity with a bilingual adults sample (Sousa & Rojjanasrirat, 2011; Vallerand, 1989). We expected acceptable internal consistency, acceptable test-retest reliability between English and French versions, and convergent responses on both versions of the instrument.

## METHOD

### PARTICIPANTS AND PROCEDURE

In this first study, the original English version of the BADS and the final French version were presented seven days apart to 147 French-English bilingual adults. Participants were recruited by Belgian and French psychology students supervised by the first and the second authors respectively in Belgium and France. All participants were informed about the study and gave their consent before starting the survey. The anonymity of data was guaranteed. The study’s protocol was approved by the ethics committee of the Psychological and Education Sciences Institute of University of Liège.^2^

At the first meeting, participants gave their informed consent and reported their current mood on three visual analogue scales (VAS), with a rating scale ranging from 1 to 10, focused on general mood, joy and sadness. Then, all participants answered a few sociodemographic questions, evaluated their reading, speaking, listening and writing skills in English on a 4-point scale, and completed one version of the BADS. At the second meeting, the participants reported their current mood in the same three VAS instruments and completed the other version of the BADS in a counterbalanced order. The order of all items was counterbalanced for each version as well. Participants with less than a total score of 12 for the self-evaluation of English language skills were excluded from the data to adhere to the Gonzalez-Reigosa approach, as modified by Vallerand and Halliwell (Vallerand & Halliwell, 1983). The original sample included 147 participants but 16 of them had total English scores under 12 and were excluded from the data. Thus, data analysis was run on 131 French-English bilingual subjects. Their mean age was 33.09 years old (SD = 13.49, range 18–64), and there were 67 women and 64 men.

### STATISTICAL AND POWER ANALYSIS

Statistical analyses computed for internal consistency relies on the calculation of Omega (ω) with 95% confidence intervals (CIs) with the MBESS package in R studio (Dunn, Baguley, & Brunsden, 2014; Kelley, 2018; Kelley & Pornprasertmanit, 2016). Omega was computed instead of the Cronbach alpha because Omega makes the assumption of multidimensionality nature of the scale which is more realistic given that only a few scales seem to be characterized by unidimentionality (Béland, Cousineau, & Loye, 2018; Socan, 2000). Omega is also associated with a lower risk of overestimation or underestimation of reliability in comparison to alpha (Dunn et al., 2014; Kelley & Pornprasertmanit, 2016).

Statistical analyses computed for test-retest reliability depend on the calculation of Pearson correlation coefficients. A correlation of between .10 and .30 corresponds to a small effect, between .30 and .50 to a medium effect and above .50 to a large effect (Cohen, 1988). To measure the responses convergence and equivalence of the two translated versions, Pearson coefficients were first computed followed by equivalence paired *t*-tests based on the two one-sided hypothesis tests with the TOSTER package in JASP (Lakens, 2017; Team, 2020). T-tests on mood scores at both evaluation times were computed to control for the effect of mood on test-retest analysis.

Descriptive analyses and correlational analyses were performed using STATISTICA 10 software (TIBCO Software Inc., 2017) with a significant criterion of .05. The de-identified data can be downloaded on the Open Science Framework on the following link: *https://osf.io/8ct7f/*. The required sample size for the Haccoun approach was *a priori* determined based on the recommendations of at least 5 subjects per item of the instrument (Nunnally & Bernstein, 1999).

## RESULTS

The 25 items had an overall ω of .85 (95 % CI [.81; .89]). The Activation subscale had an ω of .76 (95 % CI [.69; .81]). The Avoidance/Rumination subscale had an ω of .83, (95 % CI [.79; .88]). The Work/School Impairment subscale had an ω of .77, (95 % CI [.71; .83]). Finally, the Social Impairment subscale had an ω of .69 (95 % CI [.59; .79]). Test-retest reliability coefficients were acceptable with .70 for the total score, .63 for the Activation, .75 for the Avoidance/Rumination, .67 for the Work/School Impairment, and .47 for the Social Impairment. All correlations between English and French items were positive and statistically significant (all *r* > .26 *p* > .05). Equivalence paired *t*-test suggest that the lower bound test and the upper bound test were significant for all items and subscale scores. Thus, we were entitled to reject non-equivalence, and conclude that there is no meaningful difference between the French and English version of the BADS in our sample. T-tests on mood scores at both evaluation sessions revealed non-significant *p* values.

These preliminary results suggest that the scale has acceptable internal consistency, test-retest reliability and good construct validity. Because of these specific results, no adjustments were made on this version of the scale.

## STUDY 2

Study 2 was conducted to submit the final version of the French translation to Confirmatory Factor Analysis (CFA) to replicate the original factor structure in a non-clinical adult sample. We expected to find a four-factor structure corresponding to ‘Activation’, ‘Avoidance/Rumination’, ‘Work/School Impairment’ and ‘Social Impairment’. Study 2 was also conducted to examine the internal consistency and to control for the construct validity. First, to control for this construct validity, we explored the convergent validity with concurrent measures of depressive symptoms, brooding, psychological flexibility, automatic thought, and behavioral inhibition and activation system. These measures were included to follow as close as possible and thus replicate the original study. A measure of depressive symptoms is important first because activation and avoidance are good predictors of depressive symptomatology (Wagener et al., 2016), then because the negative impact on an individual’s functioning is a critical aspect of the diagnosis of depression (Chen et al., 2013; Collado et al., 2014). In addition, previous studies reported strong negative associations between activation and brooding (Nolen-hoeksema, Wisco, & Lyubomirsky, 2008), positive associations between avoidance and brooding (Giorgio et al., 2010; Moulds, Kandris, Starr, & Wong, 2007), and functioning impairments and brooding (McLaughlin, Borkovec, & Sibrava, 2007; Raes et al., 2010; Teismann et al., 2016). Psychological flexibility, defined as “the ability to contact consciously the present moment and the thoughts and feelings it contains more fully and without needless defense, and based on what the situation affords, to persist or change in behavior in the service of chosen values” (Hayes, Villatte, Levin, & Hildebrandt, 2011) has also been found to be positively associated with activation and negatively with avoidance (Kanter et al., 2007; Manos, Kanter, & Luo, 2011; Raes et al., 2010). Past studies also reported strong negative relations between activation and automatic thought (Kanter et al., 2007; Manos et al., 2011), that is, those who are less actively behaviorally also report more negative automatic thoughts. Finally, behavioral inhibition and activation systems defined as brain systems that control human behavior are generally thought to either inhibit or promote activation toward goals with previous studies reported positive relations between behavioral inhibition systems and avoidance as well as between behavioral activation systems and activation (Manos et al., 2011; Wagener, Van Der Linden, & Blairy, 2015).

Based on these previous studies, we then predicted that the total score and the Activation score would be negatively correlated with depressive symptomatology, brooding, automatic thoughts and positively correlated with psychological flexibility and behavioral activation systems. The Avoidance/Rumination score would be positively correlated with depressive symptomatology, brooding and behavioral inhibition system and negatively correlated with psychological flexibility. The Work/School Impairment and Social Impairment subscales were hypothesized to be positively correlated with depressive symptomatology and brooding.

Second, we explored the ability to predict the level of depressive symptoms based on BADS scores. We expected that the level of behavioral activation scale would be a good predictor of the level of depressive symptoms.

## METHOD

### PARTICIPANTS AND PROCEDURE

Participants were recruited via advertisements on social networks and the university messaging service. Candidates had to be French-speaking adults to be included. All participants were informed about the study and gave their consent online before starting the survey. Then they first completed sociodemographic questions followed by the random presentation of the BADS and other scales described below. The anonymity of data was guaranteed. The study’s protocol was approved by the ethics committee of the Psychological and Education Sciences Institute of University of Liège.^3^

Four hundred and eighty-five non-clinical adults completed the online survey, and 76 participants were excluded from the study because of missing data. Thus, the data analysis was run on 409 subjects. The mean age of the participants was 31.54 years (SD = 14.14, range 18–79), and they comprised 307 women and 102 men. Most participants in the sample were Belgian (84.35%) or French (11%). Approximately 49% were employed and 42.29% were students. Most of the respondents had a high school degree (34.96%), higher education of the short type of at least three years (24.69%) or higher education of the long type of at least five years as Master degree (32.27%).

The full sample had a mean Beck Depression Inventory (BDI-II) (Beck, Steer, & Brown, 1996) score of 11.82 (SD = 10.17, range 0–51), indicating a normal level of depressive symptoms. Twenty-eight percent of the participants reported one past depressive episode and 8% reported several past depressive episodes. Twenty-one participants (approximately 5%) were currently receiving treatment for a psychiatric disorder. Nine of them were taking medication for such a disorder. The demographic characteristics of the sample are reported in ***Table 1***.

**Table 1:** Demographic characteristics of the sample.


CHARACTERISTICS		M (SD) OR %

Age		31.54 (14.14)

Gender (Female/Male)		307/102

Nationality	Belgian	84.35%

	French	11%

	Luxembourger	1.46%

	Other European	3.17%

Level of education	College	3.67%

	High school	34.96%

	Higher education of the short type	24.69%

	Higher education of the long type	32.27%

	PhD Student	4.40%

Professional status	Worker	48.89%

	Student	42.29%

	Unemployed	4.65%

	Stay-at-home parent	0.48%

	Retired	3.67%

Depression	BDI-II score	11.82 (10.17)

	One past depressive episode	28.36%

	Several past depressive episodes	7.58%


### INSTRUMENTS

#### Sociodemographic questionnaire

A sociodemographic questionnaire addressed questions about nationality, age, sex, professional status, past depressive episodes and mental health.

#### Behavioral Activation for Depression Scale (BADS) (Kanter et al., 2007)

The BADS is a 25-item scale that assesses behavioral activation. Each item is rated on a 7-point scale (from 0 to 6). Four subscales have been identified: Activation (items 3, 4, 5, 7, 11, 12, 23), Avoidance/Rumination (items 8, 9, 10, 13, 14, 15, 24, 25), Work/School Impairment (items 1, 2, 6, 21, 22) and Social Impairment (items 16, 17, 18, 19, 20). A total score is computed with the sum of all items (all items are reversed except the Activation items). For the total scale, higher scores indicate higher BA. To score the subscales, no items were reverse-coded. Higher scores indicate greater activation, avoidance or impairment.

#### Beck Depression Inventory II (BDI-II) (Beck et al., 1996)

The BDI-II is a 21-item scale that assesses the severity of depressive symptoms during the last two weeks (Beck et al., 1996). Items are rated on a 4-point scale (from 0 to 3). The sum of all items constitutes a severity score for depression. Higher scores indicate more severe depression. We used the validated French version of the scale (Centre de Psychologie appliquée, 1996). In the present sample, omega (ω) for the whole scale was .92 (95 % CI [.91; .93]).

#### Ruminative Response Scale (RRS) (Treynor, Gonzalez, & Nolen-Hoeksema, 2003)

The RRS is a 22-item scale assessing rumination when respondents feel depressed, sad or discouraged. Items are rated on a 4-point scale (from 1 to 4). Two subscales have been identified, one related to Brooding (5 items) and one related to Reflection (5 items). The latter is not reported here because this aspect of rumination is more adaptive than brooding and less related to depression. Higher scores indicate higher brooding levels. We used the validated French version of the scale (Baeyens, Douilliez, & Philippot, n.d.). In the present sample, omega (ω) for the Brooding subscale was .78 (95 % CI [.75; .82]).

#### Acceptance and Action Questionnaire (AAQ-II) (Bond et al., 2011)

The AAQ-II is a 10-item scale assessing psychological flexibility in the opposition of the experiential avoidance (avoidance of thoughts, feelings, and other private events) as conceptualized by Acceptance and Commitment Therapy (Hayes, Strosahl, & Wilson, 1999). Items are rated on a 7-point scale (from 1 to 7). Some items are reversed (2, 3, 4, 5, 7, 8, 9). Higher scores indicate greater psychological flexibility and less experiential avoidance. We used the validated French version of the scale (Monestès, Villatte, Mouras, Loas, & Bond, 2009). In the present sample, omega (ω) for the whole scale was .86 (95% CI [.84; .88]).

#### Automatic Thought Questionnaire (ATQ) (Hollon & Kendall, 1980)

The ATQ is a 30-item scale assessing the valence content of automatic thoughts. Items are rated on a 5-point scale (from 1 to 5). Higher scores indicate more negative automatic thoughts. We used the validated French version of the scale (Bouvard et al., 1992). In the present sample, omega (ω) for the whole scale was .97 (95 % CI [.96; .97]).

#### Behavioral Inhibition Systems/Behavioral Activation Systems scale (BIS/BAS) (Carver & White, 1994)

The BIS/BAS scale is a 24-item scale assessing the behavioral inhibition system, which inhibits action toward goals, and three dimensions of the behavioral activation system, which promotes action toward goals. Items are rated on a 4-point scale (from 1 to 4). Several subscales have been identified: Behavioral Inhibition System-BIS (7items), BAS/Drive (4 items), BAS/Reward Responsiveness (BAS/RR) (5 items) BAS/Fun seeking (4items). In addition, there are four filler items and two items are revers. Higher BIS scores indicate greater inhibition, while higher BAS scores indicate stronger pursuit of specific goals, higher responsiveness to reward, and stronger pursuit of exciting activities, respectively. The fun seeking subscale is not reported here because this aspect of activation system is less related to everyday life activation. We used the validated French version of the scale (Caci, Deschaux, & Baylé, 2007). In the present sample, omega (ω) was .75 for the BIS (95 % CI [.72; .79], .63 for the BAS/Drive (95 % CI [.57; .69]), and .58 for the BAS/RR (95 % CI [.51; .65]).

#### Statistical and Power Analysis

To determine the accurate factor structure for the French translation of the BADS, a confirmatory factor analysis (CFA) was computed with the Lavaan package in R Studio (Rosseel, 2012). CFAs were computed on the 25 items of the French BADS. A four-factor model was tested based on the original version of the BADS and the translations. As goodness-of-fit indices to test the factor structure, the χ2 with the associated degrees of freedom, its p-value and the χ2/df were first computed. A non-significant value of χ2 and a χ2/df = 3 correspond to an acceptable fit (Cangur & Ercan, 2015). However, χ2 is sensitive to sample size (Gerbing & Anderson, 1992). Then, we computed indices more contingent on a set of cut-off scores as the Root Mean Square Error of Approximation (RMSEA), the Tucker-Lewis Index (TLI), the Comparative Fit Index (CFI) and the Standardized Root Mean square Residual (SRMR) (Bentler, 1990; Cangur & Ercan, 2015; Hu & Bentler, 1999). An RMSEA between .05 and .08, a TLI > .95, a CFI > .95, a SRMR < .10 are generally interpreted as an acceptable fit (Bentler, 1990; Schermelleh-Engel, Moosbrugger, & Müller, 2003).

Then, scale properties were computed, with internal consistency relying on the calculation of Omega (ω) with 95% confidence intervals (CIs) (Dunn et al., 2014; Kelley & Pornprasertmanit, 2016). Finally, construct validity was assessed with Pearson’s correlations and multiple linear regressions. The de-identified data can be downloaded on the Open Science Framework The de-identified data can be downloaded on the Open Science Framework on the following link: *https://osf.io/8ct7f/*. The required sample size for scale properties and Pearson’s correlations was a priori determined based on the recommendations of at least 10 subjects per item of the instrument (Nunnally & Bernstein, 1999) and based on Jackson recommendations of at least 20 subjects per estimated parameter for the CFA (Jackson, 2003).

## RESULTS

### CONFIRMATORY FACTOR ANALYSIS (CFA)

A skewness and kurtosis test indicated that multivariate normality was not respected (*p* <.001). Consequently, all CFAs were computed using a Diagonally Weighted Least Squares (DWLS) for non-normal data.

CFA revealed that the initial four-factor model do not support acceptable fit with χ2 = 1656.099, *df* = 269, *p* <.001, χ2/*df* = 6.15, RMSEA of .112, *p* <.001, 90% CI [.107; .118], TLI = .96, CFI = .96, and SRMR = .09. A significant χ2, a χ2/*df* > 3, a RMSEA > .08, and a TLI, CFI and SRMR who are acceptable but not good suggest that our data do not fit with the four-factor model as expected. Therefore, modification indices were inspected. Following the highest modification index, fit indices were calculated taking into account the suggested covariance between error variables 11 and 12. New CFA taking into account the covariance between error variables 11 and 12 support acceptable fit with χ2 =.859.320, *df* = 268, *p* < .001, χ2/*df* = 3.20, RMSEA = .074, *p* < .001, 90% CI [.068; .079], TLI = .979, CFI = .982, SRMR = .073.

The goodness-of-fit indices of the adjust 4-factor model are acceptable but not perfect. Note that rumination has sometimes been conceptualized as a cognitive avoidance behavior (Martell, Addis, & Jacobson, 2001) who may have the same function than behavioral avoidance. However, even if these two concepts may share a common function, they are somewhat different processes distinct by a behavioral action (e.g. social withdrawal) (Moulds et al., 2007).^4^ In regard to the relatively poor (even acceptable) fit of the 4-factor model, we have computed a CFA on a modified 5-factor model which fragmented the Avoidance/Rumination subscale in two distinct latent variables: Behavioral Avoidance with items 8, 9, 10, 24, and 25 (e.g. Most of what I did was to escape from or avoid something unpleasant) and Rumination with items 13, 14, and 15 (e.g. I spent a long time thinking over and over about my problems). CFA was calculated by taking into account the suggested covariance between item 11 and 12. The five-factor model was associated with χ2 = 745.293, *df* = 264, *p* <.001, χ2/*df* = 2.82, RMSEA = .067, *p* <.001, 90% CI [.061; .073], a TLI = .983, CFI =.985 and a SRMR = .069 who suggest a better model fit in comparison to the 4-factor model.

**Figure 1:**
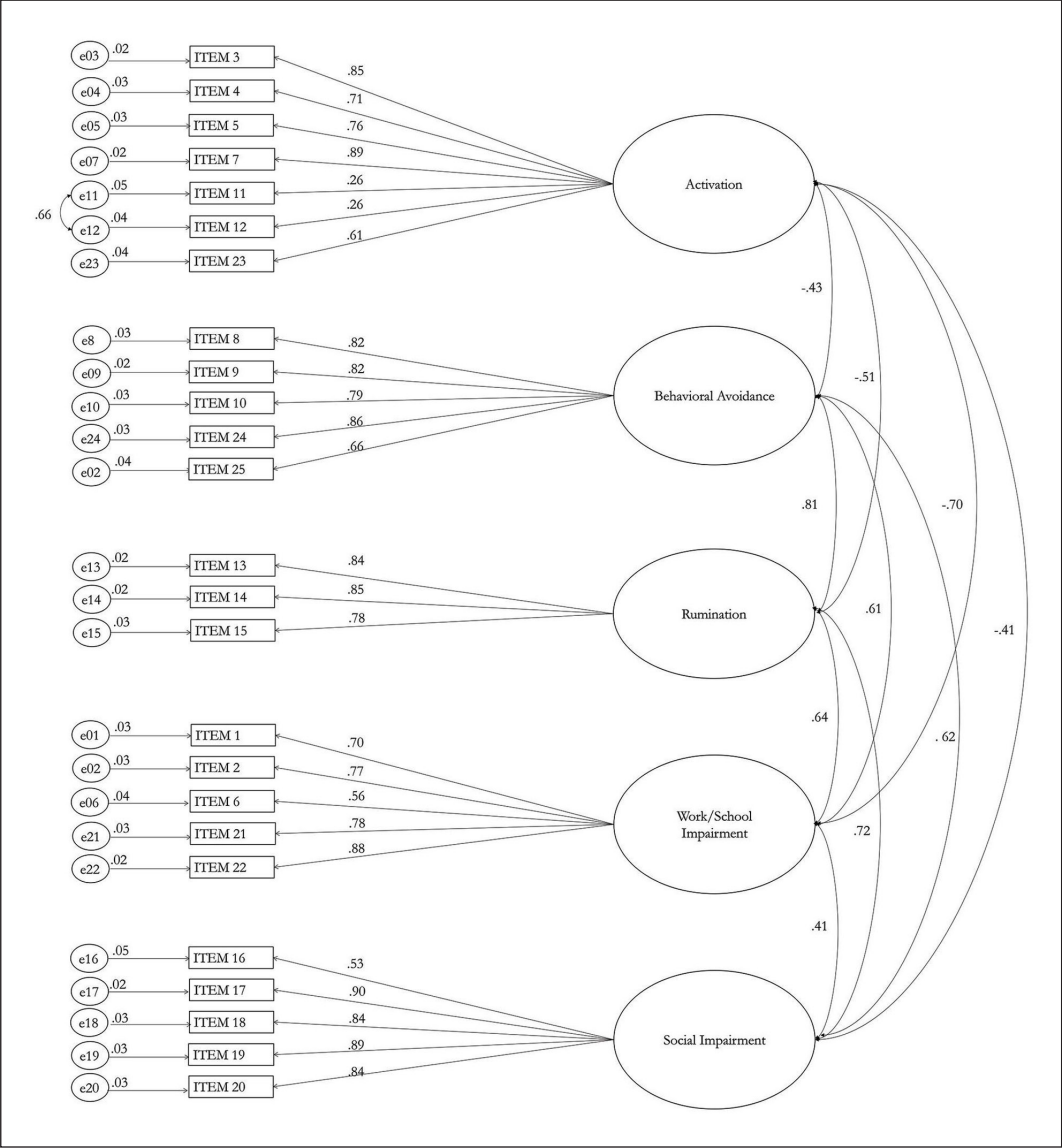
Completely standardized CFA factor solution.

Most factor loadings are in an acceptable range (λ = .53–.90) except for item 11 (λ = .26) and item 12 (λ = .26). The standardized factor solution is displayed in ***Figure 1***.

### RELIABILITY

The 25 items had an overall ω of .91 (95 % CI [.90; .92]). The Activation subscale had an ω of .83 (95 % CI [.80; .85]). The Avoidance/Rumination subscale had an ω of .89 (95 % CI [.87; .91]), the Work/School Impairment subscale had an ω of .84 (95 % CI [.81; .87]), and the Social Impairment subscale had an ω of .83 (95 % CI [.79; .86]). Behavioral Avoidance subscale had an ω of .85 (95 % CI [.82; .87]) and the Rumination had an ω of .83 (95 % CI [.79; .86]). The total and subscales scores demonstrated acceptable consistency. Inter-correlations between the total scale and all subscales are displaying in ***Table 2***. These correlations are significantly related in a consistent manner in accordance with the original scale and the literature.

**Table 2:** Correlations among BADS total and subscales of the factor structures. *Note*: All correlations are significant. (*p* < .001).


	TOTAL	ACTIVATION	AVOIDANCE	WORK/SCHOOL	SOCIAL

FOUR-FACTOR					

BADS Total	1				

Activation	.71	1			

Avoidance/Rumination	–.84	–.33	1		

Work/School Impairment	–.78	–.51	.55	1	

Social Impairment	–.66	–.26	.55	.31	1

**FIVE-FACTOR**					

Behavioral Avoidance	–.77	–.27	.95	.49	.47

Rumination	–.79	–.36	.88	.53	.55


### CONSTRUCT VALIDITY

***Table 3*** presents the Pearson’s correlations for the French BADS on one hand and the concurrent measures on the other hand. As expected, correlation analyses revealed moderate to highly significant negative correlations between the total BADS score and BDI-II, Brooding, and ATQ, and significant positive correlations with AAQ-II scores. Contrary to our expectations, the analyses revealed non-significant correlations between the total BADS score and the BAS subscales. The results are similar for Activation, except for the small but significant positive correlations with the BAS/Drive and BAS/RR. Furthermore, analyses revealed highly significant positive correlations between Avoidance/Rumination and the BDI-II, Brooding, a moderate correlation with the BIS, and a highly significant negative correlation with the AAQ-II. Finally, the Work/School Impairment and Social Impairment subscales were highly positively correlated with the BDI-II and Brooding. Additional correlations were computed given the 5-factor structure’s results with the Behavioral Avoidance subscale and the Rumination subscale and convergent measures. Correlations are similar to the pattern of correlations described for the Avoidance/Rumination subscale.

**Table 3:** Correlations among total BADS scores and subscales with concurrent measures. *Note*: BDI-II = Beck Depression Inventory – II. Brooding = Brooding subscale of Ruminative Response Scale. AAQ-II = Acceptance and Action Questionnaire – II. ATQ = Automatic Thought Questionnaire. BIS = Behavioral Inhibition Systems subscale of Behavioral Inhibition Systems and Behavioral Activation Systems scale. BAS/Drive = Behavioral Activation Systems Drive subscale of Behavioral Inhibition Systems and Behavioral Activation Systems scale. BAS/RR = Behavioral Activation Systems Reward subscale of Behavioral Inhibition Systems and Behavioral Activation Systems scale. BADS Total = BADS Total score. Activation = Activation subscale. Avoidance/R. = Avoidance/Rumination subscale. Work/S Impair. = Work/School Impairment. Social Impair. = Social Impairment. Behavioral Av. = Behavioral Avoidance.


	BDI-II	BROODING	AAQ-II	ATQ	BIS	BAS/DRIVE	BAS/RR

FOUR-FACTOR							

BADS Total	–.76**	–.57**	.69**	–.76**	–.38**	.02	.14

Activation	–.47**	–.36**	.39**	–.46**	–.24**	.13*	.21**

Avoidance/R.	.72**	.58**	–.69**	.71*	.38**	.08	–.07

Work/S Impair.	.54**	.38**	–.49**	.52*	.29**	.00	.02

Social Impair.	.54**	.39**	–.46**	.57**	.18**	–.04	–.19**

**FIVE-FACTOR**							

Behavioral Av.	.64**	.49**	–.62**	.61**	.33**	.06	–.05

Rumination	.68**	.61**	–.66**	.72**	.38**	.08	–.06


* *p* <.05. ** *p* <.01.

Simple linear regressions were applied to test if BADS total score and subscale scores significantly predict participant’s level of depressive symptoms measured by the BDI-II. Results of regressions indicated that total and subscale significantly predict BDI-II. The BADS total explains 58% of the variation of the BDI-II, *F* (1,407) = 557.62, *p* < .00; adjusted R^2^ = .58; Activation explains 22% of the variation of the BDI-II, *F* (1,407) = 117.40, *p* < .00; adjusted R^2^ = .22; Behavioral Avoidance explains 41% of the variation of the BDI-II, *F* (1,407) = 286.90., *p* < .00; adjusted R^2^ = .41), and Rumination explains 46% of the variation of the BDI-II, *F* (1,407) = 345.95, *p* < .00; adjusted R^2^ = .46. Finally, Work/School Impairment and Social Impairment each explain 29% of the variation of the BDI-II, *F* (1,407) = 164.01, *p* < .00 for the first and *F*(1,407) = 164.58, *p* < .00 for the second.

## DISCUSSION

This study reports on the psychometric results for the French version of the long-form BADS. This scale performed well in a non-clinical adult sample and similarly to the English, German and Dutch versions (Fuhr et al., 2016; Kanter et al., 2008; Kanter et al., 2007; Raes et al., 2010; Teismann et al., 2016). The preliminary investigation of the psychometric properties of the pre-final French version realized in Study 1 with a test-retest in a French-English bilingual sample revealed that the translated version had good test-retest reliability, good internal consistency and good construct validity. The psychometric properties were then examined with a larger independent sample of adults in a second study.

First, the confirmatory factor analysis revealed sufficient fit for the original four-factor computed with an adjustment that followed the highest modification index allowing for covariation of error variables associated with item 11 and 12 (‘I did things even though they were hard because they fit in with long-term goals for myself’ and ‘I did something that was hard to do but it was worth it’). The same adjustment was performed in the German validation of the scale (Teismann et al., 2016). As previously discussed by Teismann and his collaborators, the items 11 and 12 are related to effort and to the difficulty to engage in action, which is not addressed by the others indicators of activation subscale. Future research should test alternate factorial structures to improve the fit with the actual factor model using an additional latent variable related to the effort to be committed in activities. This analysis would be relevant given the central role of effort valuation in motivational theories (Wallis & Rushworth, 2013).

In addition, a CFA computed on a five-factor structure distinguishing behavioral avoidance and rumination revealed better fit indices than the original structure. Then, an independent measure to follow the course of each process during the therapy is of importance. The validation studies based on the original version of the scale did not report similar results. Previous validation studies of the BADS in other languages only attempted to replicate the original factor structure. Authors did not investigate the five-factor structure as we did. Future studies using the BADS long-form should replicate these findings to measures the fit of the five-factor structure in non-clinical and clinical depressed sample.

These results suggest that the detection of changes in activation, behavioral avoidance and rumination is possible with the actual version of the BADS (Martell et al., 2001). The reliability analyses for the total BADS and subscales revealed Omegas of at least .83, indicating good internal consistency. None of the subscales seemed to have substantially lower internal consistency than the others. In addition, the construct validity was supported by the associations between the total score and subscale scores and relevant concurrent measures of depression, brooding, psychological flexibility, negative automatic thoughts, and behavioral activation and inhibition. As in previous studies, total score and activation were negatively associated with depressive symptomatology, brooding, automatic thoughts and positively associated with psychological flexibility and behavioral activation systems for activation subscales only. In contrast, avoidance was positively associated with depressive symptomatology, brooding, and behavioral inhibition and negatively associated with psychological flexibility (Kanter et al., 2008; Kanter et al., 2007; Raes et al., 2010). Finally, impairments were positively associated with depressive symptomatology and brooding. Additional correlations computed with behavioral avoidance and rumination subscales were consistent with our main analyses.

Contrary to our expectations, the total score was not associated with behavioral activation systems measured by the BIS/BAS scale. One explanation of the lack of expected associations between the BADS total score and the BAS subscales is that the latter seem to be correlated most highly with measures of extraversion, positive affectivity and positive temperament than with anxiety, negative affectivity and avoidance, which are more similar to the measures included in our study (Jorm et al., 1999). In addition, the total score is a computation of different scores assessing activation, behavioral avoidance and rumination and not only activation. It seems that the BADS total score could be more a measure of avoidance than a measure of activation, which may explains why the BADS total score is not associated with behavioral activation systems as measured by the BAS subscale. Indeed, inter-correlations reported in ***Table 2*** revealed a higher correlation between Behavioral Avoidance or Rumination and BADS total score than between Activation and BADS total score. In the same way, the correlation between the BADS total score and the AAQ-II considered as the reverse of experiential avoidance is as high as the correlation between the Behavioral Activation and Rumination subscales and the AAQ-II. These results are consistent with past results on the original version of the scale (Kanter et al., 2007; Raes et al., 2010). Another explanation is related to the relatively low level of internal consistency of the BAS/Drive, and BAS/RR scores in this sample, suggesting that the BAS subscales may measure different qualities than expected (Carver & White, 1994). Finally, the BADS was intended to assess the degree of engagement in activities in everyday life, while the BAS/D and BAS/RR do not assess how frequently such action is encountered. In contrast, BAS subscales measure the sensitivity of systems, which react to reward or punishment. Then, these two scales are assessing somewhat different qualities that may be less related to each other than expected in our sample.

Considering the expected associations between total BADS and subscale scores and the selected relevant convergent measures of depression, brooding, psychological flexibility, negative automatic thoughts and behavioral activation and inhibition, we conclude that the French version of the BADS has good convergent validity.

Finally, simple linear regressions analyses revealed that BADS total, activation, behavioral avoidance, rumination, work and social impairments scores are each good predictors of the participant’s level of depressive symptoms. These results reflect a good criterion validity of the BADS subscales. The errors of our predictor variables are not normally distributed which is an important condition for trustworthy inference about regression coefficients. However, inferences will usually become more and more trustworthy as the sample size grows larger, even when the distribution of errors is not normal (Williams, Grajales, & Kurkiewicz, 2013). Regarding our sample size, we can considered these coefficients as relatively trustworthy. Future studies should however replicate these results and test predictive validity with a longitudinal design by measuring the ability of BADS score to predict the severity of future depression. This investigation is important because behavioral activation treatment is based on the principle that changes in activation and avoidance should mediated changes in depression.

Some limitations of this study should be discussed. First, this study involved an internet-administered tool, and findings concerning the use of online administration are mixed. Indeed, it has been reported that online and paper administration of scales result in different psychometric properties (Buchanan et al., 2005). On the other hand, some studies have found no differences between the two types of test administration (Wardenaar, Wanders, Jeronimus, & de Jonge, 2018; Zlomke, 2009). Clinicians should keep this limitation in mind when using the paper version of the BADS because the reliability and validity might differ from those of the online version, even though the preliminary results of a test-retest administered on paper are associated with good psychometric properties. A second limitation is related to self-reporting, which may be affected by memory biases whereby negative mood enhances the recall of negative information (Rusting, 1998), and also by social desirability biases (Linden, Paulhus, & Dobson, 1986). Thus, the reports may not actually reflect reality and may not reflect present the measured phenomena as expected.

Future studies should evaluate construct validity with the inclusion of divergent measures to give even more weight to construct validity of this scale. In addition, future studies should evaluate the psychometric properties, factor structure and construct validity of this scale in a clinical depressed sample.

## CONCLUSION

This French version of the BADS appears to have good psychometric properties, consistent with previous versions of the scale (English, German, and Dutch). Thus, the French translation of the BADS seems to be a reliable tool for clinicians and researchers to assess mechanisms of change and impairments in BA interventions.

## Footnotes

Items included in the French version of the short-form BADS were incorporated without translation (Wagener et al., 2015).

Reference number: 1718–80. Date of the agreement: 29-08-2018.

Reference number: 1718–80. Date of the agreement: 29-08-2018.

We thank you the reviewer for this suggestion.
